# Functional significance of NLRP3 polymorphisms in mild cognitive impairment

**DOI:** 10.1186/s12877-025-06905-6

**Published:** 2026-01-08

**Authors:** Ruonan Gao, Linda Chiu Wa Lam, Allen Ting Chun Lee, Nelson Leung Sang Tang, Suk Ling Ma

**Affiliations:** 1 Department of Psychiatry, The Chinese University of Hong Kong, Hong Kong SAR, China; 2 Department of Chemical Pathology, The Chinese University of Hong Kong, Hong Kong SAR, China; 3 School of Arts and Humanities, Tung Wah College, Hong Kong SAR, China

## Abstract

**Background:**

Nucleotide-binding domain and leucine-rich repeat (LRR)-containing family protein 3 (*NLRP3*) inflammasome is an essential component of the innate immune system and regulates inflammation. NLRP3 inflammasome has been widely studied in the pathogenesis of mild cognitive impairments (MCI) and Alzheimer’s Disease (AD). Single nucleotide polymorphisms (SNPs) of *NLRP3* gene are associated with various diseases, however the association between *NLRP3* SNPs and downstream pathway is unclear.

**Methods:**

12 tag SNPs and 2 previously reported SNPs were genotyped in 233 healthy controls (HC) and 332 MCI older adults. *NLRP3* and other inflammation-related genes expression were quantified in peripheral blood mononuclear cells (PBMC) from the older adults by quantitative PCR (qPCR). Functional studies of selected mutations were performed by luciferase assay. The older adults were followed up for 2 years to investigate the relationship between *NLRP3* SNPs and risk of cognitive decline.

**Results:**

Our study showed rs10754558 and rs7525979 were associated with an increased risk of MCI. The T allele of rs12564791 was associated with higher gene expression level of *NLRP3*, interleukin-18 (*IL-18*), *PYCARD*, and *CASP1*. rs12048215, rs10754555, and rs7525979 were associated with cognitive decline as shown by the reduction of Montreal Cognitive Assessment (MoCA) score. Functional studies showed that both rs10754558 and rs10754555 G to C mutation affected transcriptional activity. rs10754558 G to C mutation also disturbed the interaction between *NLRP3* 3’UTR and miR-425-5p. Plasma miR-425-5p expression was negatively correlated with MoCA score.

**Conclusions:**

Our study suggested that genetic variations of *NLRP3* could affect inflammatory gene expression, transcriptional activity and interaction between gene and miRNA, and therefore were associated with the risk of MCI and cognitive decline. Plasma miR-425-5p has the potential to be a biomarker for cognitive decline.

**Supplementary Information:**

The online version contains supplementary material available at 10.1186/s12877-025-06905-6.

## Introduction

Alzheimer’s Disease (AD) is a progressive neurodegenerative disease and it is the most common cause of dementia. AD is characterized by the accumulation of extracellular amyloid-beta (Aβ), intracellular neurofibrillary tangles consisting of hyperphosphorylated tau and neuronal loss [1]. The major symptoms of AD include memory loss, cognitive impairment and decline in mobility which affect both patients and their family [2]. There is currently no treatment for full recovery from AD and only symptomatic treatments are available [3]. Mild cognitive impairment (MCI) is the preclinical stage of AD. People with MCI have higher risk of developing AD [4]. Therefore, early detection and intervention is important for slowing down the disease progression.

Inflammasome is a multi-protein complex that regulates inflammation and plays an important role in the innate immune system. The NOD-like receptor pyrin domain-containing 3 (NLRP3) inflammasome is the best studied inflammasome [5]. NLRP3 consists of the sensor protein NLRP3, the adaptor protein apoptosis-associated speck-like protein containing a caspase recruitment domain (PYCARD) and the effector protein pro-caspase1. The activation of NLRP3 leads to the production and release of inflammatory cytokines interleukin-18 (IL-18) and interleukin-1 beta (IL-1β) [6]. NIMA-related kinase 7 (NEK7) is a NLRP3-binding protein that have been identified as a modulator of NLRP3 oligomerization and activation recently [7]. Several studies have demonstrated that activation of NLRP3 contributes to AD pathogenesis [8, 9]. Gene expression of *NLRP3* and downstream cytokines, IL-1β and IL-18 are up-regulated in peripheral blood mononuclear cells (PBMC) of both mild and severe AD patients [9]. Increased expression of both cytokines are also detected in cerebral temporal cortex of AD patients [10]. In APP/PS1 mouse model, *NLRP3* knockout or NLRP3 inhibition via small molecules lead to decreased Aβ accumulation, increased Aβ clearance and better cognitive function [8, 11]. Inhibition of NLRP3 also reduced the production of inflammatory cytokines [12]. These results suggest that NLRP3 participates in AD development and might be a therapeutic target.

Human *NLRP3* gene is located on chromosome 1q44, including 9 exons. Up to now, over 60 single nucleotide polymorphisms (SNPs) have been identified in *NLRP3* gene [13]. The 3’UTR SNP rs10754558 affects *NLRP3* gene expression and mRNA stability as well as its interaction with miR-146a [14–16]. The exon SNP rs35829419 influences IL-1β production in plasma [17]. Intron SNPs rs2027432 and rs4612666 can enhance the promoter activity of *NLRP3* gene [14, 18]. *NLRP3* SNPs have been associated with various diseases, including diabetes [13, 19], rheumatoid arthritis [20] and ischemic stroke [21]. Previous study also showed that *NLRP3* SNPs were associated with late-onset AD (LOAD) [22]. However, whether *NLRP3* SNPs are correlated to the susceptibility of MCI in the Chinese population is not clear.

This study investigated the relationship between *NLRP3* SNPs and the risk of MCI in the Southern Chinese population. The impact of SNPs on cognitive decline and the effect of the SNPs on *NLRP3* related gene expression were also determined. Functional studies were performed on some of the significant SNPs to elucidate the effect of these SNPs on *NLRP3* transcription and interaction with miRNA.

## Material and methods

### Participants

332 Chinese MCI older adults (41.3% men, mean age 71.38 years old, SD = 6.47, range 60–92 years) and 233 Chinese HC (40.3% men, mean age 69.06 years old, SD = 6.77, range 60–93 years) were recruited from Hong Kong. The inclusion criteria for all participants were aged 60–95, ethnic Chinese, living in the community, with no dementia (Clinical Dementia Rating (CDR) 0 or 0.5) and free of depressive symptoms and no neurological conditions that may affect cognition. CDR was used to define MCI (CDR = 0.5) or HC (CDR = 0). During recruitment, our research staff explained the procedure and obtained informed consent from the participants. The study was approved by the Clinical Research Ethics Committee of the Chinese University of Hong Kong. Clinical trial number: not applicable.

### Assessments

All participants underwent clinical and cognitive assessments at baseline interview. Including CDR and Hong Kong Montreal Cognitive Assessment (HKMoCA) [23]. CDR is a standard clinical assessment for evaluating the overall severity of cognitive impairment. HKMoCA is a locally validated cognitive screening test sensitive in detecting early memory and executive deficits. The total score of HKMoCA is 30, the higher the score, the better the cognition. A subset of participants received baseline and 2-year follow-up assessments to compare the cognitive performance over 2 years.

### SNPs selection

**Fig. 1:**
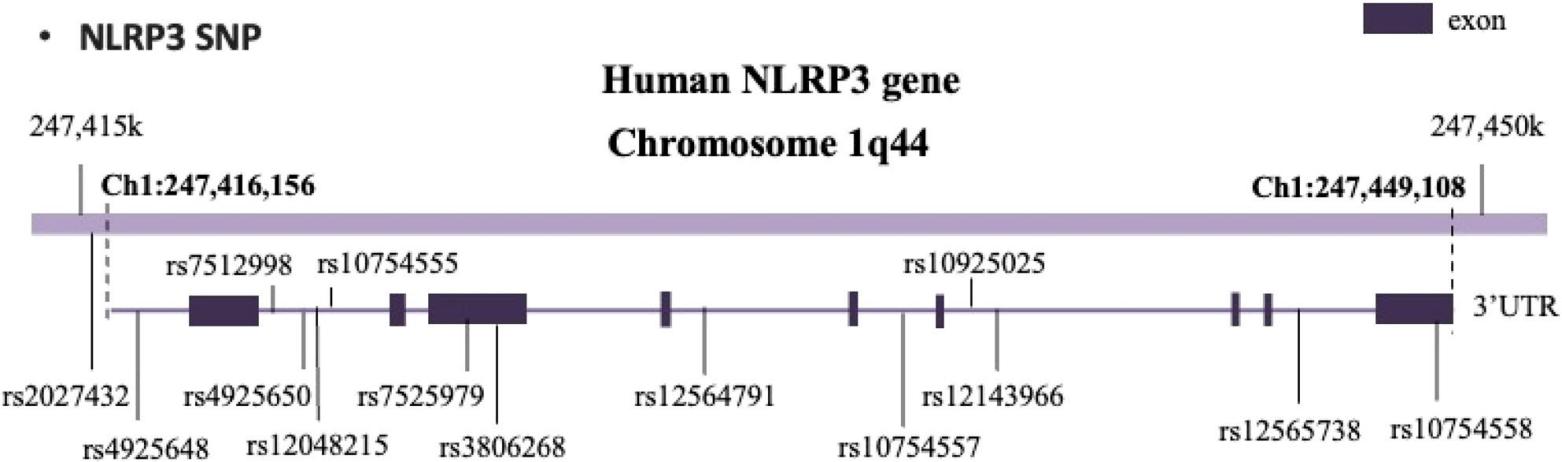
The location of NLRP3 SNPs

Tag SNPs were selected according to HapMap data of Chinese Han population (CHB) [24]. 12 tag SNPs with minor allele frequency (MAF) higher than 0.1 were chosen for genotyping. Two SNPs previously reported to be associated with AD, namely rs2027432 and rs10754558 were also included [22]. The position of the SNPs was shown in Fig. 1.

### Genotyping

Genomic DNA extraction was performed by PureLink Genomic DNA Kit (Invitrogen) from peripheral blood of MCI older adults and HC according to manufacturer’s instruction. Genotyping of *NLRP3* SNPs was performed by melting curve genotyping method. This method consisted of a set of 3 primers including 2 allele-specific primers and a common reverse primer. The 2 allele-specific primers containing additional sequence of different length at 5′ end would produce polymerase chain reaction (PCR) products with different melting temperature due to differences in GC composition. A long 5′ GC sequence (5′-GCGGGCAGGGCGG-3′) and a short 5′ GC sequence (5′-GCGGGC-3′) was added to the allele-specific primers respectively. Genotypes at the SNP can be inferred from the different melting temperature of the PCR products. Melting curve analysis was performed by LC480 (Roche). Primers used in genotyping were listed in Supplementary Table 1.

### RNA extraction, reverse transcription and quantitative PCR (qPCR)

Total RNA was extracted from PBMC by Trizol LS and bromochloropropane (BCP), followed by isopropanol precipitation and 75% ethanol wash. miRNA was extracted from plasma by the same method with 1 µl glycogen (R0551, Thermo Fisher). Total RNA reverse transcription was performed by PrimeScript RT Reagent Kit (RR037A, Takara). q PCR was performed by TB Green Premix Ex Taq II (Tli RNase H Plus) kit (RR820A, Takara) in a volume of 10 µl, including 5 µl 2X SYBR Master Mix, 0.4 µl forward and reverse primer, 0.05 µl dUTP, 2.15 µl H2O and 2 µl cDNA. miRNA reverse transcription was performed by MicroRNA first-strand synthesis and miRNA quantitation kits (638315, TaKaRa). miRNA qPCR was performed in a volume of 10 µl, including 5ul 2X SYBR Master Mix, 0.4 µl forward and reverse primer, 3.2 µl H2O and 1 µl cDNA. Each sample was run in duplicate. The relative expression of target genes was normalized to *UBC* for mRNA and *U6* for miRNA and calculated by 2 △△Ct method. qPCR was performed by LC480. Primers used in qPCR were listed in Supplementary Table 2.

### Plasmids

Gene fragments containing target SNPs were amplified by KAPA HiFi Hotstart ReadyMix PCR Kit (Roche). After purification, the PCR products were ligated to NheI digested pGL3-promoter vector by In-Fusion Cloning Kit (Takara) and transformed to Stbl3. Site-directed mutagenesis at selected SNPs was performed by KAPA HiFi. All purified plasmid sequences were confirmed by Sanger sequencing.

### Cell culture and transfection

HEK293FT cells were maintained in DMEM medium with 10% fetal bovine serum (FBS) and 1% penicillin/streptomycin in humidified incubator at 37 ℃ with 5% CO_2_. Transfection was performed using Lipofectamine 2000 when cells reached 50% confluency. To measure transcriptional activity, 500 ng pGL3-promoter vector and 50 ng pRL-CMV vector were transfected into HEK293FT cells for 48 h. For miRNA interaction, 10 nM miR-425-5p mimics or control mimics (Genepharma, China) were transfected into HEK293FT together with luciferase vectors.

### Luciferase reporter assay

Gene fragments of NLRP3 with wild type or mutant nucleotide was cloned into pGL3-promoter vector. 500 ng constructed pGL3-promoter vector or empty pGL3-promoter vector were cotransfected with 50 ng Renilla luciferase reporter vector pRL-CMV into HEK293FT cells. Dual-luciferase Reporter Assay kit (Promega) was used to measure firefly and renilla luciferase activity 48 h after transfection. Relative luciferase activity was calculated as firefly luciferase activity divided by renilla luciferase activity. Each group was performed in triplicate.

### Statistical analysis

Deviations from the Hardy–Weinberg equilibrium (HWE) for genotypes at individual loci were assessed by using the Pearson chi-squared test with one degree of freedom. Comparison between two groups was carried out by Student’s t-test and three groups by one-way ANOVA with Bonferroni post hoc test. Differences in genotype and allelic distribution of *NLRP3* SNPs between MCI and HC were tested by Chi-square test. Dominant model was applied to increase the precision [25]. Logistic regression was used to assess the strength of association between *NLRP3* SNPs and MCI by calculating the odds ratios (ORs) and 95% confidence intervals (CIs), adjusting for age and gender. Correlation analysis was used to study inflammatory gene levels. Data were analyzed by GraphPad Prism 10.0 and SPSS 29.0. P-value < 0.05 was considered as statistically significant.

## Results

### Demographic characteristics

The demographic characteristics of the studied population were presented in Table 1. No significant differences were observed in the distribution of sex and ApoE genotypes between MCI and HC. MCI older adults were older (*p* < 0.001) and with lower MoCA score (*p* < 0.001) than HC at baseline.

**Table 1:** Demographic characteristics of the participants at baseline

	MCI	HC	*P* value
Sex (F/M)	195/137	139/94	0.826
Age (SD)	71.38 (6.47)	69.06 (6.77)	< 0.001*
MoCA (SD)	21.38 (4.08)	25.12 (3.40)	< 0.001*
ApoE2 (%)	48 (16.05)	33 (19.54)	0.431
ApoE3 (%)	198 (66.22)	146 (70.53)	
ApoE4 (%)	53 (17.73)	28 (13.53)	

*MCI* Mild cognitive impairment, *HC* Healthy controls, *F* Female, *M* Male, *SD* Standard deviation, *MoCA* Montreal cognitive assessment, *ApoE* Apolipoprotein E

### Association of *NLRP3* SNPs with MCI

The genotype distribution of rs7525979 (*p* = 0.046) and rs10754558 (*p* = 0.049) were significantly different between MCI older adults and HC. Since the frequency of rs7525979 TT genotype was only 1.5%, collapsing the genotypes as dominant model will increase the statistical power [26]. Dominant model analysis was also performed for rs10754558. Difference in allelic frequency was also found in rs7525979 (*p* = 0.014). rs7525979 T allele carriers and rs10754558 C allele carriers were associated to significantly higher risk of developing MCI (Table 2). For other selected SNPs, there was no significant difference in the genotype distribution between MCI and HC. Education was analyzed as covariate for a subgroup of participants (153 HC and 205 MCI) due to limited information available. Logistic regression results showed rs10754558 (*p* = 0.030) and rs7525979 dominant model (*p* = 0.046) distribution differed between MCI and HC.

**Table 2:** Genotype and allele distribution of *NLRP3* SNPs in older adults with MCI and HC

	MCI (%)	HC (%)	*P* value	95% CI
rs7525979 C/T				
Genotype				
CC	251 (76.1)	197 (84.2)		
CT	74 (22.4)	36 (15.4)	0.046*	
TT	5 (1.5)	1 (0.4)		
TT + CT vs. CC (dominant model)			0.019*	1.087–2.583
Allele				
C	576 (87.3)	430 (91.9)	0.014*	1.103–2.469
T	84 (12.7)	38 (8.1)		
rs10754558 C/G				
Genotype				
CC	127 (38.6)	70 (29.8)	0.049*	
CG	150 (45.6)	131 (55.7)		
GG	52 (15.8)	34 (14.5)		
CC vs. CG + GG (dominant model)			0.030*	1.044–2.163
Allele				
C	404 (61.4)	271 (57.7)	0.207	0.918–1.486
G	254 (38.6)	199 (42.3)		

*95% CI* 95% confidence interval

*p<0.05

### Association between *NLRP3* SNPs and inflammation related gene expression

**Fig. 2:**
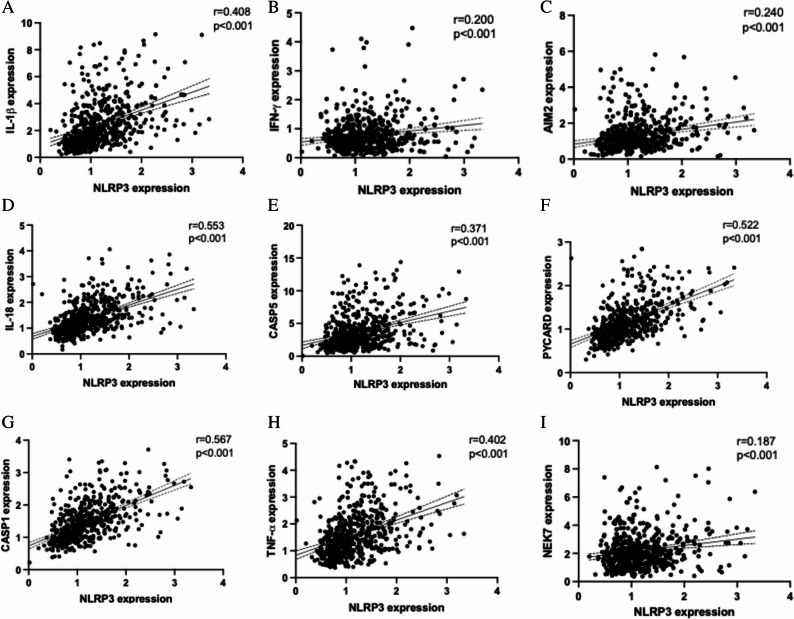
*NLRP3* gene expression was positively correlated with other inflammatory gene expression. Correlation between gene expression of *NLRP3* with gene expression of (**A**) *IL-1β* (**B**) *IFN-γ* (**C**) *AIM2* (**D**) *IL-18* (**E**) *CASP5* (**F**) *PYCARD* (**G**) *CASP1* (**H**) *TNF-α* and (**I**) *NEK7* in PBMC

*NLRP3* mRNA level was positively correlated with *IL-1β*, *IL-18*, *IFN-γ*, *AIM2*, *CASP1*, *CASP5*, *PYCARD*, *TNF-α* and *NEK7* gene expression in PBMC (*p* < 0.001) after controlling for age and sex (Fig. 2).

**Fig. 3:**
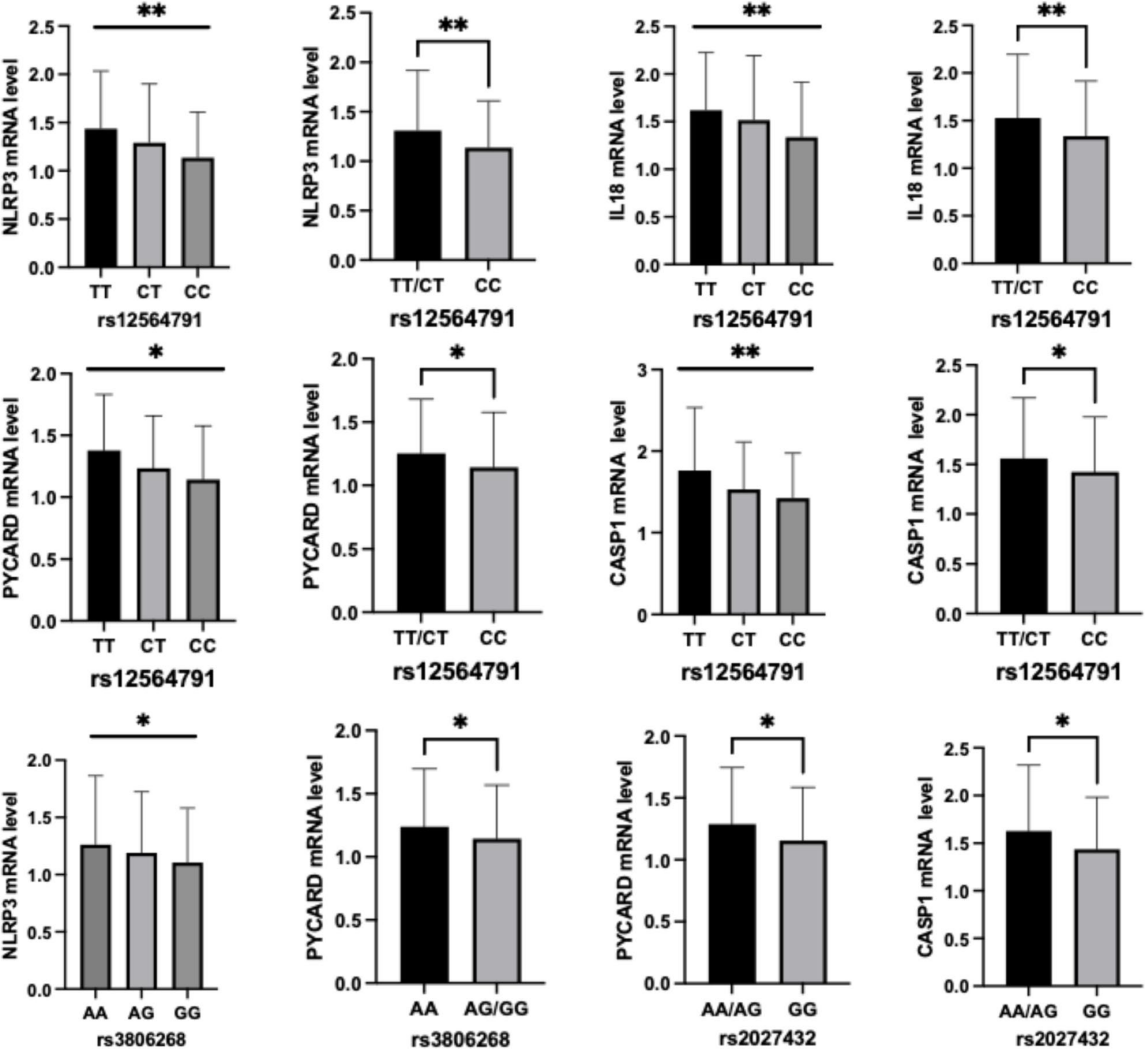
Association between *NLRP3* SNPs and inflammation related gene expression. *p < 0.05,**p < 0.01

The gene expression level of inflammatory related genes were quantified in MCI older adults and HC. There was no significant difference in the selected gene expression between MCI older adults and HC. The correlation between gene expression level and *NLRP3* SNPs was also investigated. After adjusting for age and gender, rs12564791 was associated with the gene expression levels of *NLRP3* (*p* = 0.001), *IL-18* (*p* = 0.001), *PYCARD* (*p* = 0.005), *CASP1* (*p* = 0.006) and *IL-1β* (*p* = 0.032). rs12564791 TT genotype showed the highest gene expression and CC genotype showed the lowest gene expression among these four genes. Similar trend was also found in the dominant model of rs12564791. rs3806268 was associated with higher *NLRP3* (*p* = 0.015) and *PYCARD* gene expression (*p* = 0.024). rs2027432 GG genotype was associated with higher *PYCARD* (*p* = 0.034) and *CASP1* (*p* = 0.017) gene expression (Fig. 3). For other tested SNPs, no association between SNP genotypes and inflammatory gene expression was found.

### Association of *NLRP3* SNPs with cognitive decline

We next investigated whether *NLRP3* SNPs were associated with cognitive decline. 266 participants joined the 2-year follow-up study, including 161 MCI and 105 HC at baseline study. 13 HC progressed to MCI and 1 HC progressed to AD during the 2 years. 4 MCI older adult progressed to AD at the 2-year follow-up and the remaining older adults remained stable. In the current study, MoCA score drop of 2 points or higher was considered as cognitive decline. The demographics characteristics of the follow-up participants were listed in Table 3. There were no differences in sex, age and ApoE genotype between participants with MoCA score drop of 2 points or higher and MoCA scored drop less than 2 points. Participants with MoCA decrease ≥ 2 showed significantly lower MoCA score in follow-up study compared with HC (Table 3).

**Table 3:** Demographic characteristics of the participants at follow-up

	MoCA decrease ≥ 2 (%)	MoCA decrease < 2 (%)	*P* value
Sex (F/M)	26/11	138/90	0.266
Age (SD)	74.81 (8.00)	72.26 (6.03)	0.070
MoCA (SD)	21.54 (3.10)	25.48 (3.74)	< 0.001
ApoE2 (%)	3 (9.09)	32 (15.02)	
ApoE3 (%)	21 (63.64)	149 (69.96)	0.179
ApoE4 (%)	9 (27.27)	32 (15.02)	

*F* Female, *M* Male, *SD* Standard deviation, *MoCA* Montreal cognitive assessment

rs10754555 CC genotype were associated with a higher risk of cognitive decline (MoCA score decrease > = 2) at 2-years follow-up (*p* = 0.048) (Table 4). No other SNP was found to be associated with MoCA score decline during the 2-years follow-up. The follow-up results showed that *NLRP3* SNP rs10754555 might have predictive value for cognitive decline.

**Table 4:** Association between *NLRP3* SNPs with change in MoCA score

	MoCA decrease > = 2 (%)	MoCA decrease < 2 (%)	*P* value	95% CI
rs10754555 C/G				
Genotype				
CC	18 (45.0)	67 (29.3)	0.140	
CG	18 (45.0)	130 (56.8)		
GG	4 (10.0)	32 (13.9)		
CC vs. CG + GG (dominant model)			0.048^*^	1.017–4.095
Allele				
C	54 (67.5)	264 (57.6)	0.098	0.923–2.525
G	26 (32.5)	194 (42.4)		

*95% CI* 95% confidence interval

*p < 0.05

### rs10754555 affected transcriptional activity

**Fig. 4:**
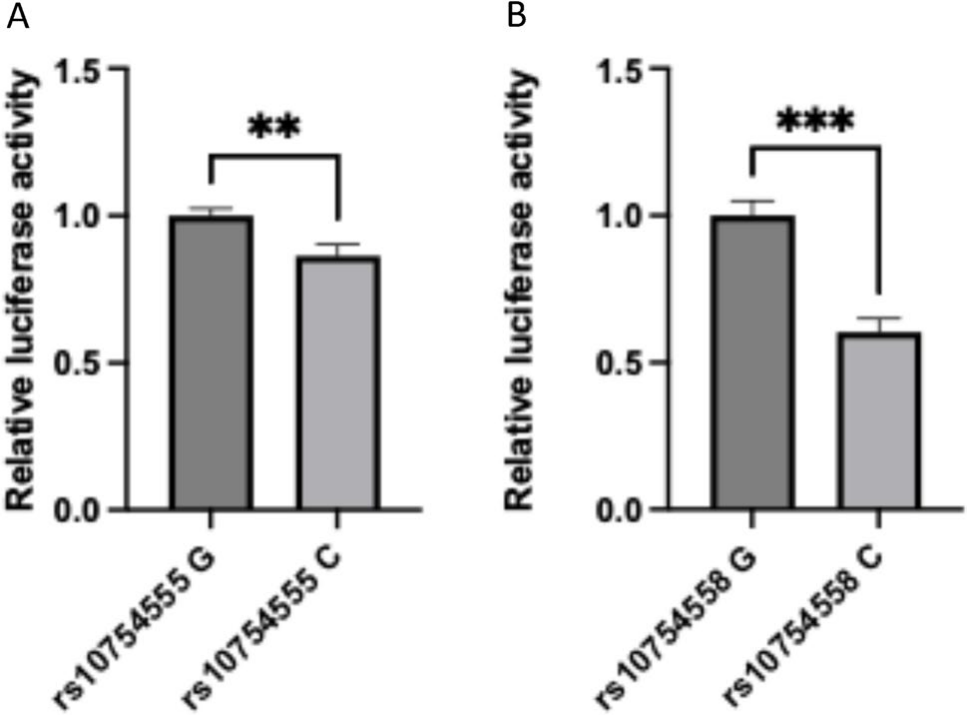
rs10754555 and rs10754558 affected transcriptional activity. (**A) **transcriptional activities of rs10754555 G/C were detected using dual-luciferase reporter assay in HEK293FT cells; (**B**) transcriptional activities of rs10754558 G/C were detected using dual-luciferase reporter assay in HEK293FT cells. *p < 0.05, ***p < 0.001

Rs10754555 locates in the intronic region of *NLRP3* and rs10754558 locates on 3’-UTR of *NLRP3*, we next investigated whether the G to C mutation of both SNPs enhance gene transcription. 300 bp mRNA secondary structure was predicted by RNAfold Web Server (http://rna.tbi.univie.ac.at/cgi-bin/RNAWebSuite/RNAfold.cgi) and the result showed the RNA secondary structure containing C nucleotides of both SNPs was associated with a higher free energy and lower stability, when compared to the structure with G nucleotides. G-to-C mutation might change the structure of *NLRP3* folding and therefore affected the downstream function. To confirm this prediction, pGL3-promoter vectors containing ~ 1000 bp *NLRP3* intron sequence with rs10754555 G or C were transfected into HEK293FT cells and luciferase reporter assay was performed. Vectors with G allele showed significantly higher luciferase activity, suggesting higher transcriptional activity associated with G allele of these two SNPs (Figs. 4A-B).

### rs10754558 affected interaction with miR-425-5p

**Fig. 5:**
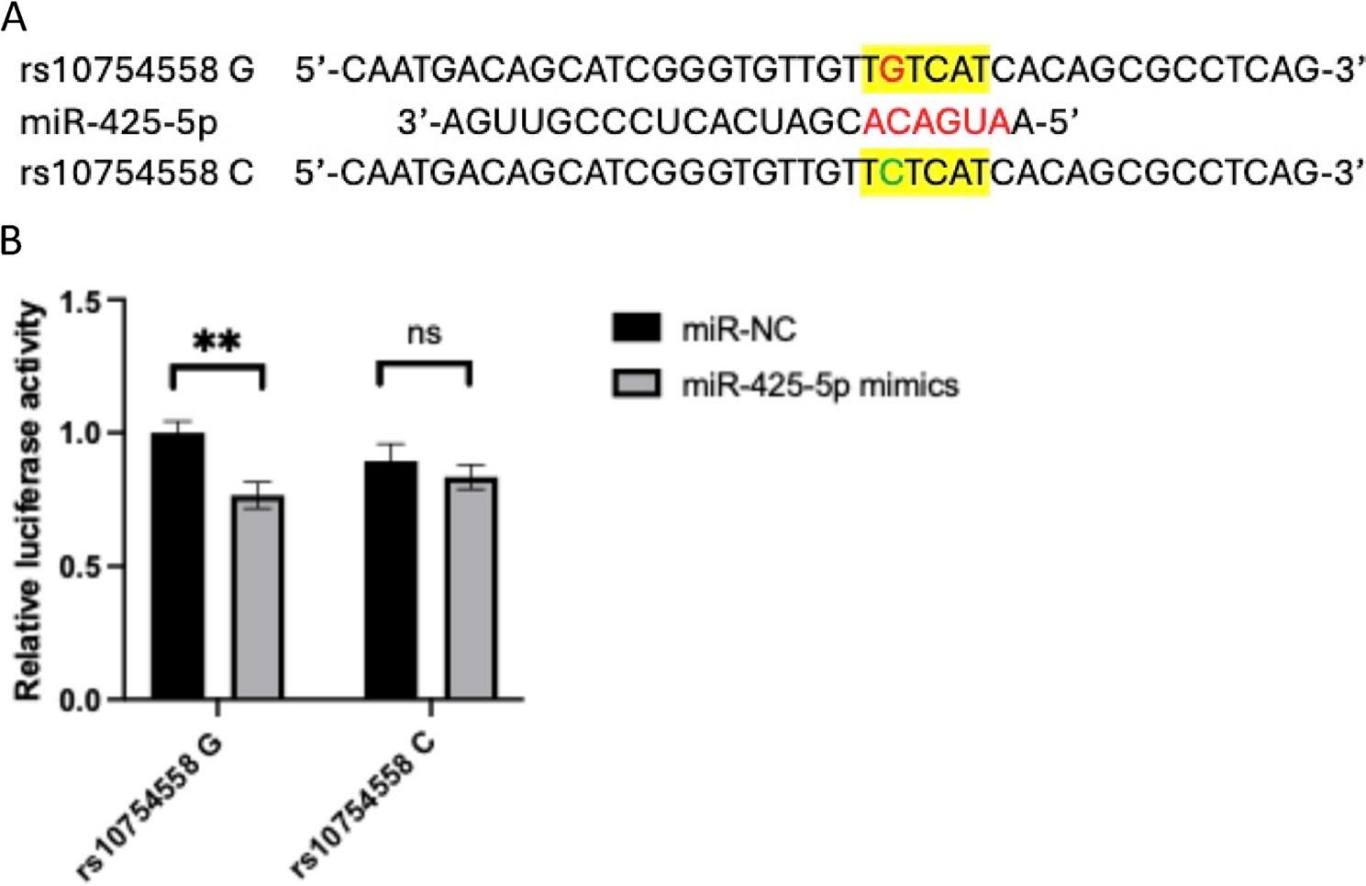
rs10754558 affects interaction with miR-425-5p. (**A)** rs10754558 G to C mutation was predicted to disturb the binding between *NLRP3* 3’-UTR and miR-425-5p; (**B**) The binding activities of rs10754558 G/C with miR-425-5p were determined by dual-luciferase reporter assay in HEK293FT cells. **p < 0.01

As rs10754558 locates within the 3’-UTR of *NLRP3* gene, we further investigated whether it could affect the interactions with miRNAs. A SNP-miRNA interaction prediction tool, miRNASNP-v3 [27], showed that rs10754558 is within the binding site of miR-425-5p and the G-to-C mutation disrupted this interaction (Fig. 5A). To validate this interaction, pGL3-promoter vectors containing *NLRP3* 3’-UTR sequence with rs10754558 G or C were co-transfected with miR-425-5p mimics or miRNA negative control into HEK293FT cells for 48 h. The dual luciferase assay showed that miR-425-5p significantly reduced the luciferase activity in cells transfected with vector containing rs10754558 G allele. No obvious changes were found in cells transfected with rs10754558 C allele (Fig. 5B). This result indicated that *NLRP3* is a target gene of miR-425-5p and rs10754558 G-to-C mutation affected the binding of miR-425-5p to *NLRP3* 3’-UTR.

### miR-425-5p negatively correlated with MoCA

**Fig. 6:**
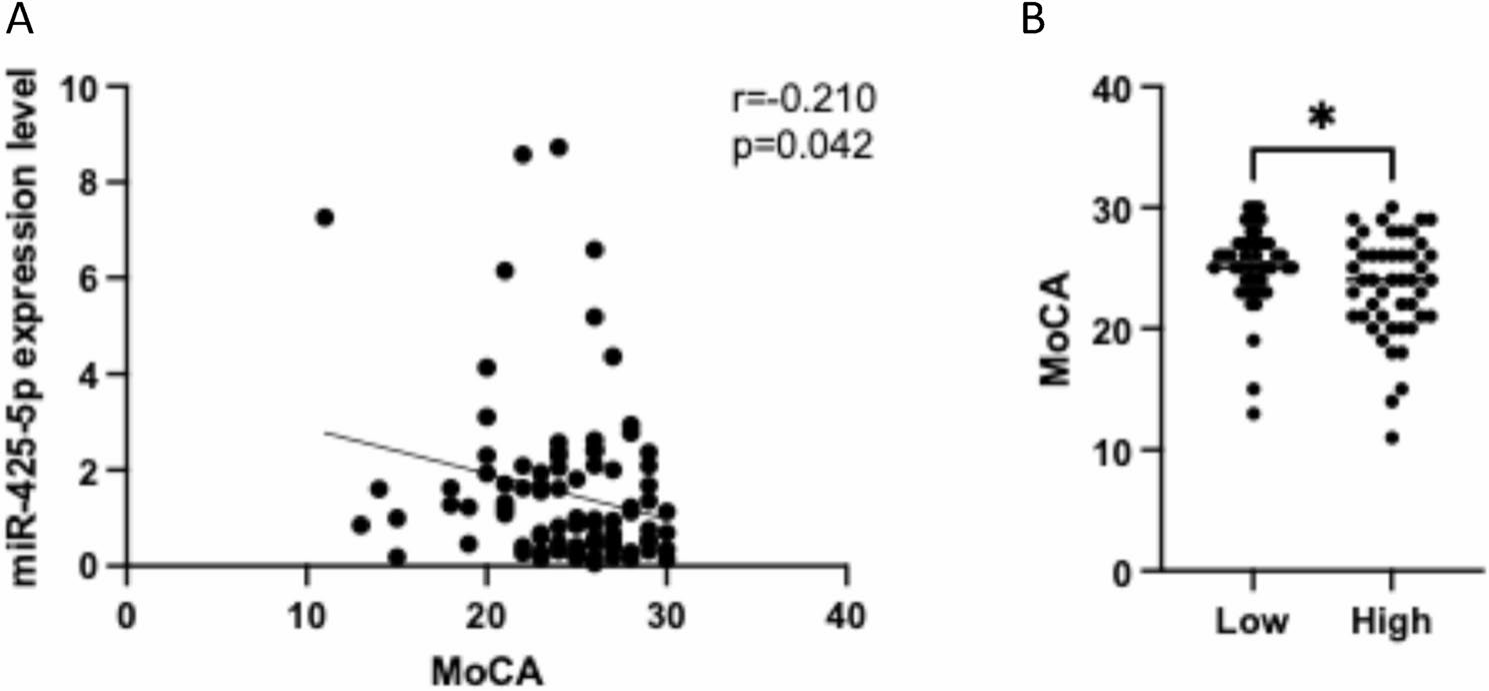
miR-425-5p negatively correlated with MoCA. (**A)** miR-425-5p expression negatively correlated with MoCA score; (**B**) MoCA score was significantly different between high or low miR-425-5p expression group. *p < 0.05

Plasma RNA was extracted from 45 MCI older adults and 45 age- and gender-matched HC and reverse transcription was performed by adding Poly(A) tail to the 3’ end of miRNA. Correlation analysis showed that plasma miR-425-5p level had a slight negative correlation trend with MoCA score (*r*=-0.210, *p* = 0.042) (Fig. 6A). When using the median of miR-425-5p expression level to define high and low expression groups, MoCA score were significantly different between the two groups. High miR-425-5p expression group was associated to lower average MoCA score (*p* = 0.011) (Fig. 6B).

## Discussion

Increasing evidence demonstrate the important role of inflammation in MCI and AD pathogenesis. NLRP3 is a key inflammasome participating in inflammation and inflammatory cytokines production. Genetic variations of *NLRP3* have been identified in multiple inflammatory related disease, including inflammatory bowel disease, periodontitis, asthma, primary gouty arthritis and autoimmune thyroid disease [28–32]. Previous studies have found that rs2027432 was associated with LOAD risk and rs10754558 was associated with LOAD only in *ApoE* ε4 carriers [22]. Our study identified the associations between *NLRP3* SNPs rs7525979 and rs10754558 and the onset of MCI in Chinese. Individuals carrying at least one T allele of rs7525979 or one C allele of rs10754558 had higher risk of developing MCI than those without copies of the risk allele. In addition, rs10754558 could affect *NLRP3* mRNA level and increase mRNA stability [14, 16]. rs10754558 G to C mutation was demonstrated to alter the interaction between *NLRP3* and miR-146a-5p, which further confirmed that this SNP may be functional [15]. Our study also showed that rs10754558 might regulate *NLRP3* gene expression and affect the interaction with miR-425-5p. rs7525979, a synonymous SNP located in exon 3, have been shown to affect the efficiency of *NLRP3* translation, impact NLRP3 protein stability, ubiquitination state, and solubility [33]. Synonymous mutations leading to rare codons were generally considered to translate more slowly [34]. The deregulated translation rate may cause protein misfolding, which was widely recognized to affect proteinopathies like AD [33]. In our finding, rs7525979 was associated with MCI risk and cognitive decline. The current study also identified that another synonymous variant rs3806268 was associated with the gene expression of *PYCARD*. The result might be explained by the altered translation efficacy and protein folding. This in turn influenced protein function and downstream pathways which further result in disease progression. As the duration of this study was only two years, it was difficult to observe CDR changes; therefore, MoCA score decreases equal or over 2 points was considered as cognitive decline in this study [35]. The 2-year follow up results showed that rs10754555 was associated with decline in MoCA score decline. rs10754555 G to C mutation affected transcriptional activity, which may result in altered gene expression and NLRP3 function and therefore influence cognition.

Although introns consist of 90% of gene sequence, most functional mutations are found in exons [36]. Recent studies have highlighted the impact of intron variants to regulate splicing efficiency and abnormal splicing, including exon skipping and intron inclusion [36, 37]. Intronic SNPs could also influence allele imprinting, gene expression and cell apoptosis [38]. Most of the SNPs investigated in our study are located in intron regions. rs12564791 was associated with the gene expression level of *NLRP3* and *IL-18*. rs2027432 was associated with the gene expression of *PYCARD* and *CASP1*. NLRP3 consists of the sensor NLRP3, the adaptor PYCARD and the effector pro-caspase1. Upon inflammasome activation, NLRP3 recruits PYCARD and forms a large filament molecule ASC speck. Assembled ASC speck recruits pro-caspase 1 and enables caspase 1 self-cleavage and activation. During the assembly process, the interaction between NEK7 and NLRP3 increases which is essential for ASC speck formation and caspase 1 activation [7]. Caspase 1 further cleaves the cytokine precursors, pro-IL-18 and pro-IL-1β, to their active form IL-18 and IL-1β. The intron SNPs of *NLRP3* affected gene expression of NLRP3 inflammasome components and also downstream cytokines, indicating their potential role in modulating inflammasome assembling and function.

Previous study showed gene expression level of *NLRP3* but no other related molecules was upregulated in stimulated monocytes of MCI older adults [9]. However, no significant difference of any inflammation gene expression between MCI older adults and HC was found in our study. This may be due to different methodology designs as our study directly assessed mRNA level in PBMC. In the initial stage of the disease, inflammation genes may start to express abnormally but not yet reach obvious changes without extra stimulations. The expression of *NLRP3* was positively correlated with all other inflammation genes, indicating the potential assembly step as the disease progresses.

miRNAs are small, single-stranded, non-coding RNA that play crucial roles in regulating gene expression, as well as cell proliferation, apoptosis, invasion and migration. miRNAs have been implicated in AD pathogenesis and the expression of several miRNAs differed between AD patients and healthy individuals in brain, cerebrospinal fluid, and blood [39]. miRNAs achieve their function by binding to the 3’-UTR of target genes and further lead to the upregulation or downregulation of target genes. Therefore, mutations within 3’-UTR may affect the interactions between target genes and miRNAs. rs10754558 G to C mutation was predicted to be a binding site of miR-425-5p. Our functional study confirmed that the 3’UTR SNP rs10754558 might affect the interaction between *NLRP3* 3’-UTR with miR-425-5p. miR-425-5p has been shown to be upregulated in plasma of AD patients, which might be a potential biomarker [40]. miR-425-5p may participate in AD by interacting with BACE1 and HSPB8 and further regulate BACE1 activity, promote tau phosphorylation and cell apoptosis [40, 41]. miR-425-5p was negatively associated with blood-based inflammation markers C-reactive protein and fibrinogen, indicating its role in inflammation [42]. Our results showed that plasma miR-425-5p expression was negatively correlated with MoCA score, which was in line with previous study. However, our results did not show any difference between MCI older adults and HC regarding miR-425-5p expression level, probably due to the small sample size.

There were a few limitations in the current study. The distribution of genotypes and alleles varies among different ethnicities. The current study included only Chinese participants and the findings may not be applicable to other ethnic groups. Future research should be replicated in other ethnic groups to validate the association between *NLRP3* SNPs and the risk of MCI. Dropout rate is inevitable in studies involving older participants [43]. Only parts of participants took part in the follow up study, which might lead to bias in the results. Older adults quitted from the study were more likely to have worsen cognitive function [44]. The 2-year interval between baseline and follow-up study was relatively short and it is expected only a small proportion of the participants presented with significant cognitive decline, which might affect the power of the statistical analysis. Long-term studies should be performed and data can be collected at multiple time points to investigate the progression of disease over time. The sample size for plasma miRNA quantification experiment was limited, despite the samples were age- and sex-matched. Future studies with more samples will be needed to confirm the potential of miR-425-5p as a biomarker for cognitive decline. Melting curve genotyping was used to genotype *NLRP3* SNPs in this study. While melting curve genotyping method is easy and cheap, ambiguities are rapidly increasing as more and more SNPs are discovered [45]. The more superior next generation sequencing can be used for higher capacity and resolution. Although age was corrected in this study, the effect of inflamm-aging should be taken into consideration. Aging of human is accompanied by increased level of inflammatory markers [46]. Age correction may be insufficient to account for the non-linear changes and accumulation of inflammation over time, leaving unmeasurable biases. In the current study, subtype of MCI was unknown. Future studies may consider to investigate the association between the risk of specific MCI groups and *NLRP3* SNPs.

In summary, our study suggested that *NLRP3* SNPs are associated with the risk of MCI and cognitive decline in Chinese, probably be affecting inflammatory gene expression, transcriptional activity and miRNA interaction. These results provided new insights into the genetic effect of NLRP3 inflammasome on MCI and treatment against inflammation might be a potential therapeutic strategy.

## Supplementary Information

Supplementary Material 1.

## Data Availability

All data generated or analyzed during this study are included in this article. Part of the data was previously presented in conference and published as abstract in the conference proceedings in Alzheimer’s and Dementia [47]. Further enquiries can be directed to the corresponding author.
