# Supplementary material for: Functional significance of NLRP3 polymorphisms in mild cognitive impairment
Source: BMC Geriatr. 2026 Jan 8;26:173. doi: 10.1186/s12877-025-06905-6 (PMC12882300; doi:10.1186/s12877-025-06905-6)
Supplement: Supplementary file 1 — Supplementary Material 1. [file 12877_2025_6905_MOESM1_ESM.docx]

**Table S1. Primers used in genotyping**

| SNP | Primer type | Primer sequence |
| --- | --- | --- |
| rs2027432 | AS1 | 5’-**GCGGGC**CAGTTCTGTAGAAAGGTGGTA-3’ |
|  | AS2 | 5’-**GCGGGCAGGGCGGC**CAGTTCTGTAGAAAGGTGGTG-3’ |
|  | CP | 5’-GGCACAGAGAAGCTAGAATAAGCAG-3’ |
| rs10754558 | AS1 | 5’-GCGGGCCAGCATCGGGTGTTGTTC-3’ |
|  | AS2 | 5’-GCGGGCAGGGCGGCCAGCATCGGGTGTTGTTG-3’ |
|  | CP | 5’-CCAAGAGGAACATCCTCTAACTG-3’ |
| rs12565738 | AS1 | 5’-GCGGGCATCCACACCCTTGCTAT-3’ |
|  | AS2 | 5’-GCGGGCAGGGCGGCATCCACACCCTTGCTAC-3’ |
|  | CP | 5’-CGACTGAAATGGGGAAGAATCAGGT-3’ |
| rs4925648 | AS1 | 5’-GCGGGCGTTCTAAACCCCTCGGT-3’ |
|  | AS2 | 5’-GCGGGCAGGGCGGCGTTCTAAACCCCTCGGC-3’ |
|  | CP | 5’-ATAGATGCATAAACTGAGTCTTAGGGGG-3’ |
| rs7512998 | AS1 | 5’-GCGGGCGACCCTGCTTTGAATTCTT-3’ |
|  | AS2 | 5’-GCGGGCAGGGCGGCGACCCTGCTTTGAATTCTC-3’ |
|  | CP | 5’-CCGTATGATTCAGAAATTTCACTTCTG-3’ |
| rs10754557 | AS1 | 5’-GCGGGCCATGGTGGAGCGTGA-3’ |
|  | AS2 | 5’-GCGGGCAGGGCGGCCATGGTGGAGCGTGG-3’ |
|  | CP | 5’-TTCCATAGAGATGGCAGAATGTCA-3’ |
| rs10754555 | AS1 | 5’-GCGGGCGGGAGGTCAGAAACCAC-3’ |
|  | AS2 | 5’-GCGGGCAGGGCGGCGGGAGGTCAGAAACCAG-3’ |
|  | CP | 5’-TGAGTAGCTGGGACTACAGGTGA-3’ |
| rs12564791 | AS1 | 5’-GCGGGCCGTTTGTTTGTCTTGCTGTT-3’ |
|  | AS2 | 5’-GCGGGCAGGGCGGCCGTTTGTTTGTCTTGCTGTC-3’ |
|  | CP | 5’-GATGGGTACTAAGATGTCTGTCCAG-3’ |
| rs4925650 | AS1 | 5’-GCGGGCAGGGCGGCCTCTTTTCCCCTCTCACG-3’ |
|  | AS2 | 5’-GCGGGCCTCTTTTCCCCTCTCACA-3’ |
|  | CP | 5’-GAAAGCAAACAGTTTGCCTCCACC-3’ |
| rs12143966 | AS1 | 5’-GCGGGCAGGGCGGCCTATTGTCAATACCTAACCTGG-3’ |
|  | AS2 | 5’-GCGGGCCTATTGTCAATACCTAACCTGA-3’ |
|  | CP | 5’-GGCCGCTCTGTTTGTGATACTTTATT-3’ |
| rs12048215 | AS1 | 5’-GCGGGCAGGGCGGCCAGCAGTGTGGGTGTAG-3’ |
|  | AS2 | 5’-GCGGGCCAGCAGTGTGGGTGTAA-3’ |
|  | CP | 5’-CGGCTGCACACTGCTGTCTGTT-3’ |
| rs3806268 | AS1 | 5’-GCGGGCGATGATGTTGGACTGGGCA-3’ |
|  | AS2 | 5’-GCGGGCAGGGCGGCAGCAGGTGCTCAGTGAAG-3’ |
|  | CP | 5’-GAACAGATAGTCAAACCTGTCTTGG-3’ |
| rs7525979 | AS1 | 5’-GCGGGCCTGAGCCTGTGCACACT-3’ |
|  | AS2 | 5’-GCGGGCAGGGCGGCCTGAGCCTGTGCACACC-3’ |
|  | CP | 5’-GGATTGTTTTCCCAATCCCTGCCG-3’ |
| rs10925025 | AS1 | 5’-GCGGGCAGCAGGTGCTCAGTGAAA-3’ |
|  | AS2 | 5’-GCGGGCAGGGCGGCAGCAGGTGCTCAGTGAAG-3’ |
|  | CP | 5’-GGGCTCTTCCGGCTCCTTCACAG-3’ |

**Table S2. Primers used in qPCR**

| Gene | Forward primer sequence | Reverse primer sequence |
| --- | --- | --- |
| UBC | 5’-CGTCCGTCGCCAGCCGGGATTTGGGTCG-3’ | 5’- CGACGCAGCCCACGAAGATCTGCATTGTCAAGT-3’ |
| NLRP3 | 5’-GTGTGAAACGCTCCAGCATC-3’ | 5’-CAGCAGCTGACCAACCAGAG-3’ |
| IL18 | 5’-TGCAGTCTACACAGCTTCGG-3’ | 5’-TAATTTAGATTCAAGCTTGCCAAAG-3’ |
| IL1β | 5’-CAGAAGTACCTGAGCTCGGC-3’ | 5’-GGTCGGAGATTCGTAGCTGG-3’ |
| PYCARD | 5’-CCTCAGTCGGCAGCCAAG-3’ | 5’-TCCTCCACCAGGTAGGACTC-3’ |
| CASP1 | 5’-ACATCCCACAATGGGCTCTG-3’ | 5’-TCCTCCACCAGGTAGGACTC-3’ |
| CASP5 | 5’-GTGCTGTACAAAGAGACAGAGG-3’ | 5’-AGATGTTTGTCCAGCCACGTT-3’ |
| IFNγ | 5’-CAGCTCTGCATCGTTTTGGG-3’ | 5’-TCTGTCACTCTCCTCTTTCCAA-3’ |
| AIM2 | 5’-GGCTGATCCCAAAGTTGTCAG-3’ | 5’-CCGCCCCAGCATTTTGAATC-3’ |
| NEK7 | 5’-CCACTGGGATGGTAAAACTTG-3’ | 5’-AAGGACTTTGTAATGCAGCCAT-3’ |
